# Association Between Cardiorespiratory Fitness and Cancer Incidence and Cancer-Specific Mortality of Colon, Lung, and Prostate Cancer Among Swedish Men

**DOI:** 10.1001/jamanetworkopen.2023.21102

**Published:** 2023-06-29

**Authors:** Elin Ekblom-Bak, Emil Bojsen-Møller, Peter Wallin, Sofia Paulsson, Magnus Lindwall, Helene Rundqvist, Kate A. Bolam

**Affiliations:** 1 Department of Physical Activity and Health, The Swedish School of Sport and Health Sciences, Stockholm, Sweden; 2 Research Department, HPI Health Profile Institute, Stockholm, Sweden; 3 Department of Psychology, University of Gothenburg, Stockholm, Sweden; 4 Department of Laboratory Medicine, Karolinska Institutet, Stockholm, Sweden; 5 Department of Neurobiology, Care Sciences and Society, Karolinska Institutet, Stockholm, Sweden

## Abstract

**Importance:**

Cardiorespiratory fitness (CRF) levels appear to be an important risk factor for cancer incidence and death.

**Objectives:**

To examine CRF and prostate, colon, and lung cancer incidence and mortality in Swedish men, and to assess whether age moderated any associations between CRF and cancer.

**Design, Setting, and Participants:**

A prospective cohort study was conducted in a population of men who completed an occupational health profile assessment between October 1982 and December 2019 in Sweden. Data analysis was performed from June 22, 2022, to May 11, 2023.

**Exposure:**

Cardiorespiratory fitness was assessed as maximal oxygen consumption, estimated using a submaximal cycle ergometer test.

**Main Outcomes and Measures:**

Data on prostate, colon, and lung cancer incidence and mortality were derived from national registers. Hazard ratios (HRs) and 95% CIs were calculated using Cox proportional hazards regression.

**Results:**

Data on 177 709 men (age range, 18-75 years; mean [SD] age, 42 [11] years; mean [SD] body mass index, 26 [3.8]) were analyzed. During a mean (SD) follow-up time of 9.6 (5.5) years, a total of 499 incident cases of colon, 283 of lung, and 1918 of prostate cancer occurred, as well as 152 deaths due to colon cancer, 207 due to lung cancer, and 141 deaths due to prostate cancer. Higher levels of CRF (maximal oxygen consumption as milliliters per minute per kilogram) were associated with a significantly lower risk of colon (HR, 0.98, 95% CI, 0.96-0.98) and lung cancer (HR, 0.98; 95% CI, 0.96-0.99) incidence, and a higher risk of prostate cancer incidence (HR, 1.01; 95% CI, 1.00-1.01). Higher CRF was associated with a lower risk of death due to colon (HR, 0.98; 95% CI, 0.96-1.00), lung (HR, 0.97; 95% CI, 0.95-0.99), and prostate (HR, 0.95; 95% CI, 0.93-0.97) cancer. After stratification into 4 groups and in fully adjusted models, the associations remained for moderate (>35-45 mL/min/kg), 0.72 (0.53-0.96) and high (>45 mL/min/kg), 0.63 (0.41-0.98) levels of CRF, compared with very low (<25 mL/min/kg) CRF for colon cancer incidence. For prostate cancer mortality, associations remained for low (HR, 0.67; 95% CI, 0.45-1.00), moderate (HR, 0.57; 95% CI, 0.34-0.97), and high (HR, 0.29; 95% CI, 0.10-0.86) CRF. For lung cancer mortality, only high CRF (HR, 0.41; 95% CI, 0.17-0.99) was significant. Age modified the associations for lung (HR, 0.99; 95% CI, 0.99-0.99) and prostate (HR, 1.00; 95% CI, 1.00-1.00; *P* < .001) cancer incidence, and for death due to lung cancer (HR, 0.99; 95% CI, 0.99-0.99; *P* = .04).

**Conclusions and Relevance:**

In this cohort of Swedish men, moderate and high CRF were associated with a lower risk of colon cancer. Low, moderate, and high CRF were associated with lower risk of death due to prostate cancer, while only high CRF was associated with lower risk of death due to lung cancer. If evidence for causality is established, interventions to improve CRF in individuals with low CRF should be prioritized.

## Key Points

**Question:**

Is there an association between cardiorespiratory fitness and colon, lung, and prostate cancer incidence and mortality in Swedish men?

**Findings:**

In this cohort of 177 709 men followed up for a mean of 9.6 years, higher cardiorespiratory fitness was associated with a lower risk of colon cancer incidence. A lower risk of death from lung and prostate cancer was also noted.

**Meaning:**

The findings of this study suggest that cardiorespiratory fitness may have a potentially important role in reducing the risk of developing and dying from certain cancers in men.

## Teaser

This cohort study compares the incidence and risk of death associated with lung, colon, and prostate cancer in men with varying levels of cardiorespiratory fitness.

## Introduction

Cancer is the second leading cause of death worldwide,^1^ and cancer cases are estimated to increase and reach 21.4 million by 2030.^2^ The main reasons for this increase are a combination of growing and aging populations and medical advances, leading to a greater number of cancers being detected. Many risk factors contributing to the global cancer burden are modifiable behavioral factors.^3^ Physical activity is a well-known modifiable risk factor for an increasing number of cancer types.^4^

Although physical activity (PA) is a behavior that involves movement of the skeletal muscles, cardiorespiratory fitness (CRF) is a more objective reflection of an individual’s current level of moderate-to-vigorous intensity PA^5^; however, CRF is also influenced (25% or more) by a person’s genetics.^6^ Regular PA has been associated with a lower risk of several cancer types,^4,7^ yet few studies have investigated the influence of CRF on cancer incidence or mortality. Of these studies, analysis of the association between CRF and both cancer incidence and mortality in the same study, reported by cancer type, and conducted in large population samples are lacking in existing studies.^8,9,10,11^ Moreover, previous studies have typically only examined main effects, thereby ignoring important interaction analyses of, for example, when in adulthood associations between CRF and cancer outcomes are particularly strong or weak. As increasing age is one of the strongest risk factors for several cancer forms^12^ and CRF levels tend to decrease with older age,^13^ the associations between CRF and cancer incidence and mortality risk may vary depending on when in life CRF is assessed.^14^

With the concerning number of people who are estimated to receive a cancer diagnosis in the coming decades, it is imperative that researchers investigate potential preventable risk factors to develop targeted prevention strategies. Therefore, the aims of this study were to investigate whether there is an association between midlife CRF and colon, lung, and prostate cancer incidence and mortality in men and study any moderation of the associations by age.

## Methods

In this prospective cohort study, data were retrieved from the health profile assessment (HPA) database, managed by the HPI Health Profile Institute (Stockholm, Sweden). Health profile assessments have been carried out in Swedish health services throughout Sweden since the 1970s, and the results of these HPAs have been stored in a central database since 1982. Each assessment comprises measurement of body mass and height; a submaximal cycle ergometer test to assess CRF; a questionnaire with items on PA habits, lifestyle, and perceived health; and an in-depth interview with an HPA coach. Participation was optional, free of charge for the individual, and offered to all employees working for a company or organization connected to occupational or other health services. The Health Profile Institute is responsible for the development and standardization of methods, education of data collection staff, and administration of the central database. The study was approved by the Stockholm Ethics Review Board and adhered to the Declaration of Helsinki.^15^ Men provided informed consent before participating in the HPA. All data were deidentified and analyzed at a group level. This study follows the Strengthening the Reporting of Observational Studies in Epidemiology (STROBE) reporting guideline for observational studies.

From October 1982 until December 2019 (month of follow-up of outcome data in national registers), data from 180 764 men (age, 18-75 years) with a first-time HPA and valid estimate of CRF were stored in the central HPA database. Of these, a total of 3055 records were missing data for covariates included in the full model (educational level, age, smoking, diet, comorbidity, and body mass index [BMI]); hence, 177 709 men were included in the analyses. eFigure 2 in Supplement 1 provides a flow chart of included and excluded men. Excluded individuals were somewhat younger (mean age, 40.8 vs 41.9 years; *P* < .001) but had similar CRF (mean, 36.9 vs 36.7 mL/min/kg; *P* = .37) as included participants. Furthermore, men already diagnosed with colon, prostate, or lung cancer at the time of HPA participation were excluded in the relevant analysis of each cancer (Table 1).

**Table 1.** Characteristics and Outcome Data of the Total Study Population and by Cardiorespiratory Fitness Group

Characteristic	Total	Cardiorespiratory fitness, mL/min/kg			
		Very low (≤25)	Low (>25-35)	Moderate (>35-45)	High (>45)
No.	177 709	19 606	64 901	58 736	34 466
Age, mean (SD), y	42 (11)	49 (10)	45 (11)	40 (11)	35 (9)
University degree, No. (%)	41 166 (23)	2626 (13)	11 940 (18)	14 877 (25)	11 723 (34)
BMI, mean (SD)	26.2 (3.8)	30.0 (4.8)	27.1 (3.5)	25.3 (2.9)	24.0 (2.4)
BMI ≥30, No. (%)	24 819 (14)	8551 (44)	12 176 (19)	3671 (6)	421 (1)
Never daily smoking, No. (%)	146 448 (82)	15 621 (80)	52 556 (81)	48 848 (83)	29 423 (85)
Very poor/poor alcohol habits, No. (%)	9119 (5)	961 (5)	3240 (5)	2986 (5)	1932 (5)
Very poor/poor diet, No. (%)	14 715 (8)	2218 (11)	5715 (9)	4482 (8)	2300 (7)
Number of comorbidities, No. (%)^a^					
0	164 932 (93)	17 091 (87)	59 073 (91)	55 416 (94)	33 352 (97)
1	12 111 (7)	2336 (12)	5507 (9)	3183 (5)	1085 (3)
2	647 (0)	172 (1)	313 (0.5)	134 (0)	28 (0)
3	19 (0)	7 (0)	8 (0)	3 (0)	1 (0)
Absolute V̇o_2_max, mean (SD), L/min	3.08 (0.76)	2.13 (0.38)	2.67 (0.43)	3.26 (0.47)	4.06 (0.59)
Relative V̇o_2_max, mean (SD), mL/min/kg	36.7 (9.9)	22.1 (2.3)	30.4 (2.8)	39.6 (2.8)	52.0 (6.0)
Incidence outcomes					
Colon cancer cases, No. (%)	499 (0)	106 (1)	248 (0)	112 (0)	33 (0)
Excluded due to colon cancer prior to HPA, No.	90	14	54	19	3
Colon cancer follow-up, mean (SD), y	9.6 (5.5)	8.6 (5.2)	9.4 (5.4)	9.8 (5.5)	10.0 (5.6)
Lung cancer cases, No. (%)	283 (0)	68 (0)	129 (0)	71 (0)	15 (0)
Excluded due to lung cancer before HPA, No.	10	5	3	2	0
Lung cancer follow-up, mean (SD), y	9.6 (5.5)	8.6 (5.2)	9.4 (5.4)	9.8 (5.5)	10.0 (5.6)
Prostate cancer cases, No. (%)	1918 (1)	300 (2)	910 (1)	572 (1)	136 (0)
Excluded due to prostate cancer prior to HPA, No.	383	84	175	105	19
Prostate cancer follow-up, mean (SD), y	9.5 (5.5)	8.6 (5.2)	9.3 (5.4)	9.8 (5.5)	10.0 (5.6)
Mortality outcomes					
Colon cancer deaths, No. (%)	152 (0)	28 (0)	79 (0)	35 (0)	10 (0)
Lung cancer deaths, No. (%)	207 (0)	52 (0)	97 (0)	52 (0)	6 (0)
Prostate cancer deaths, No. (%)	141 (0)	39 (0)	68 (0)	30 (0)	4 (0)
Follow-up mortality, mean (SD), y	9.6 (5.5)	8.6 (5.2)	9.4 (5.4)	5.5 (9.8)	5.6 (10.0)

Abbreviations: BMI, body mass index (calculated as weight in kilograms divided by height in meters squared); HPA, health profile assessment; V̇o_2_max, maximal oxygen consumption.

^a^ Including hypertension, cardiovascular disease, diabetes, and other cancer forms. Percentages may not total 100 because of rounding.

### Assessment of Cardiorespiratory Fitness

The standardized submaximal Åstrand cycle ergometer test was used to estimate maximal oxygen consumption (V̇o_2_max).^16^ To standardize the assessment, participants were requested to refrain from the following before the test: vigorous PA the day before, consuming a heavy meal 3 hours before, smoking/snuff use 1 hour before, and stress. In adult populations, a previous validation study showed small, nonsignificant mean differences at a group level (−0.07 L/min; 95% CI, −0.21 to 0.06 L/min) between estimated V̇o_2_max using the Åstrand test and directly measured V̇o_2_max during maximal effort tests on a treadmill, with an absolute error and coefficient of variation similar to other submaximal tests (standard error of estimate, 0.48 L/min; coefficient of variation, 18.1%).^17^ In the analyses in our study, CRF was used as a continuous variable as well as after stratification into 4 groups. Stratification was first based on the cutoff for men (10 metabolic equivalents of task, approximately 35 mL/min/kg) from the landmark work of Blair et al,^18^ used as the middle cutoff, and then on 1 higher (45 mL/min/kg) and 1 lower (25 mL/min/kg) arbitrarily chosen as cutoffs to create 4 groups: very low CRF (≤25 mL/min/kg), low CRF (>25-35 mL/min/kg), moderate CRF (>35-45 mL/min/kg), and high CRF (>45 mL/min/kg).

### Cancer Incidence and Mortality Surveillance

The Swedish National Patient Registry and National Cause of Death Registry were used to retrieve data on first-time cancer-specific events and mortality. Analyses were conducted at an individual level by linking the study data to the unique Swedish personal identity number. For the incidence analyses, all participants were followed up from their HPA to the first cancer-specific event, death, or December 31, 2019. For the mortality analyses, all participants were followed up from their HPA to their cancer-specific death, death by other causes, or December 31, 2019. The *International Statistical Classification of Diseases and Related Health Problems, 10th Revision* (*ICD-10*) codes used for cancer incidence and cancer-specific death were C18 for colon cancer, C34 for lung cancer, and C61 for prostate cancer.

### Assessments of Covariates

Covariates were chosen based on available evidence of their influence on cancer and mortality and were proposed in a working model graph (eFigure 1 in Supplement 1). The highest level of education attained (length of education: <9 years to postgraduate education) at the time of the HPA was drawn from Statistics Sweden by linking the participant’s personal identity number. Smoking, diet, and alcohol habits were all self-reported using the following statements. I smoke at least 20, 11 to 19, or 1 to 10 cigarettes per day; occasionally; or never). I consider my diet, regarding both meal frequency and nutritional content to be very poor, poor, neither good nor bad, good, or very good. I consider my alcohol habits, from a health perspective, to be very poor, poor, neither good nor bad, good, or very good. Body mass was assessed with a calibrated scale to the nearest 0.5 kg with the patient wearing lightweight clothing. Body height was assessed using a wall-mounted stadiometer to the nearest 0.5 cm. Body mass index was subsequently calculated as weight in kilograms divided by height in meters squared. Comorbidity was ascertained using the Swedish National Patient Registry. A comorbidity variable (ranging from a possible score of 0-4) was created by summing the number of the following comorbidities each person had before or after the HPA: hypertension (*ICD-10* codes I10-I13), cardiovascular disease (*ICD-10* codes I20-I25, I60-I69), diabetes (*ICD-10* codes E10-E14), and other cancers (*ICD-10* codes C00-C99).

### Statistical Analysis

Data analysis was performed from June 22, 2022, to May 11, 2023. General linear modeling was used to compare estimated V̇o_2_max (means adjusted for age) between incident and mortality cases compared with noncases. Each of the 6 outcomes (colon, lung, and prostate cancer incidence and death) and CRF were compared on a continuous scale and were analyzed using Cox proportional hazards regression models with natural cubic splines. Knots were set at 25, 35, and 45 mL/min/kg to match the cutoffs used for the analyses of the 4 CRF groups. Reference levels were set at 22 mL/min/kg, which represents the mean estimated V̇o_2_max among participants below the lowest knot. To study the associations between CRF in 4 groups and the outcomes, Cox proportional hazards regression modeling was used, estimating hazard ratios (HRs) and 95% CIs. The proportional hazards assumption was checked using scaled Schoenfeld residuals, with 0 slopes on functions of time for all outcomes except for prostate cancer incidence. Because of this, we included an interaction between time and estimated V̇o_2_max, with no change in the results. Three models were used and adjusted for by an increasing number of variables for each model. The models were built differently to include BMI as a variable in the evaluation of colon and prostate cancer and smoking in the evaluation of lung cancer. For colon and prostate cancer, model 1 included age and performed year of HPA; model 2 additionally adjusted for length of education, smoking, diet habits, and comorbidity; and model 3 additionally adjusted for BMI. For lung cancer, model 1 included age and performed year of HPA; model 2 additionally adjusted for length of education, diet habits, comorbidity, and BMI; and model 3 additionally adjusted for smoking. Sensitivity analyses was performed for model 3 analyses by dropping incident and mortality cases that occurred 2 years or less after the HPA (Table 2). To test for interactions between CRF and age at HPA, an interaction term was introduced in the Cox proportional hazards regression analyses and significant interactions were defined, with 2-sided, unpaired testing, as *P* < .05 for the interaction term. Prevented fraction of the population was calculated for the CRF groups with significant associations with decreased risk for cancer incidence and/or death.^19^ It is interpreted as the theoretical reduction in incidence or mortality that would hypothetically occur due to exposure to higher CRF (in the 3 higher groups >25-35, >35-45, >45 mL) compared with if the whole population was exposed for only the lowest levels (≤25 mL). Statistical analyses were performed using SPSS, version 27 (SPSS Inc) and R, version 4.2.1, with Tidyverse, Survival, Splines, and Patchwork packages (R Foundation for Statistical Computing).

**Table 2.** Association Between CRF and Incidence and Mortality in Colon Cancer, Lung Cancer, and Prostate Cancer^a^

Variable	Cancer incidence										Cancer mortality									
	Crude model		Model 1		Model 2		Model 3		Model 3, 2-y exclusion		Crude model		Model 1		Model 2		Model 3		Model 3, 2-y exclusion	
	HR (95% CI)	*P* value for trend	HR (95% CI)	*P* value for trend	HR (95% CI)	*P* value for trend	HR (95% CI)	*P* value for trend	HR (95% CI)	*P* value for trend	HR (95% CI)	*P* value for trend	HR (95% CI)	*P* value for trend	HR (95% CI)	*P* value for trend	HR (95% CI)	*P* value for trend	HR (95% CI)	*P* value for trend
**Colon cancer**																				
Cases/total (n/No.)	499/177 709	NA	499/177 709	NA	499/177 709	NA	499/177 709	NA	451/177 571	NA	152/177 709	NA	152/177 709	NA	152/177 709	NA	152/177 709	NA	142/177 609	NA
Very low CRF, ≤25 mL/min/kg	1 [Reference]	<.001	1 [Reference]	<.001	1 [Reference]	<.001	1 [Reference]	0.007	1 [Reference]	0.004	1 [Reference]	<.001	1 [Reference]	0.02	1 [Reference]	0.09	1 [Reference]	0.16	1 [Reference]	0.3
Low CRF, >25-35 mL/min/kg	0.62 (0.49-0.78)		0.83 (0.66-1.05)		0.85 (0.68-1.07)		0.93 (0.74-1.18)		0.91 (0.71-1.17)		0.73 (0.48-1.13)		0.97 (0.63-1.50)		1.04 (0.67-1.61)		1.07 (0.68-1.67)		1.08 (0.68-1.72)	
Moderate CRF, >35-45 mL/min/kg	0.29 (0.22-0.38)		0.59 (0.45-0.77)		0.61 (0.47-0.81)		0.72 (0.53-0.96)		0.69 (0.51-0.95)		0.33 (0.20-0.55)		0.66 (0.40-1.10)		0.75 (0.45-1.26)		0.79 (0.45-1.36)		0.83 (0.47-1.47)	
High CRF, >45 mL/min/kg	0.14 (0.09-0.21)		0.48 (0.32-0.72)		0.51 (0.33-0.77)		0.63 (0.41-0.98)		0.58 (0.36-0.93)		0.16 (0.08-0.32)		0.51 (0.24-1.09)		0.60 (0.28-1.29)		0.64 (0.29-1.43)		0.74 (0.32-1.67)	
**Lung cancer**																				
Cases/Total (n/No.)	283/177 709	NA	283/177 709	NA	283/177 709	NA	283/177 709	NA	254/177 670	NA	207/177 709	NA	207/177 709	NA	207/177 709	NA	207/177 709	NA	193/177 685	NA
Very low CRF, ≤25 mL/min/kg	1 [Reference]	<.001	1 [Reference]	0.005	1 [Reference]	0.007	1 [Reference]	0.46	1 [Reference]	0.54	1 [Reference]	<.001	1 [Reference]	0.002	1 [Reference]	0.002	1 [Reference]	0.17	1 [Reference]	0.11
Low CRF, >25-35 mL/min/kg	0.50 (0.37-0.67)		0.71 (0.53-0.95)		0.70 (0.51-0.94)		0.82 (0.60-1.11)		0.83 (0.60-1.15)		0.48 (0.34-0.68)		0.71 (0.50-0.99)		0.68 (0.48-0.96)		0.80 (0.57-1.14)		0.77 (0.54-1.10)	
Moderate CRF, >35-45 mL/min/kg	0.28 (0.20-0.39)		0.67 (0.48-0.94)		0.66 (0.46-0.96)		0.91 (0.63-1.31)		0.98 (0.67-1.44)		0.27 (0.18-0.39)		0.67 (0.45-1.00)		0.64 (0.42-0.98)		0.88 (0.58-1.35)		0.91 (0.59-1.40)	
High CRF, >45 mL/min/kg	0.10 (0.06-0.17)		0.46 (0.26-0.82)		0.45 (0.24-0.82)		0.72 (0.39-1.34)		0.63 (0.31-1.26)		0.05 (0.02-0.12)		0.27 (0.11-0.64)		0.24 (0.10-0.59)		0.41 (0.17-0.99)		0.21 (0.06-0.72)	
**Prostate cancer**																				
Cases/Total (n/No.)	1918/177 709	NA	1918/177 709	NA	1918/177 709	NA	1918/177 709	NA	1695/177 103	NA	141/177 709	NA	141/177 709	NA	141/177 709	NA	141/177 709	NA	137/177 322	NA
Very low CRF, ≤25 mL/min/kg	1 [Reference]	<.001	1 [Reference]	0.01	1 [Reference]	0.12	1 [Reference]	0.59	1 [Reference]	0.62	1 [Reference]	<.001	1 [Reference]	0.001	1 [Reference]	0.004	1 [Reference]	0.009	1 [Reference]	0.01
Low CRF, >25-35 mL/min/kg	0.81 (0.71-0.92)		1.18 (1.03-1.34)		1.15 (1.01-1.31)		1.11 (0.97-1.27)		1.14 (0.99-1.32)		0.42 (0.28-0.63)		0.61 (0.41-0.92)		0.66 (0.45-0.99)		0.67 (0.45-1.00)		0.66 (0.43-0.99)	
Moderate CRF, >35-45 mL/min/kg	0.53 (0.46-0.61)		1.33 (1.15-1.53)		1.26 (1.09-1.45)		1.18 (1.02-1.38)		1.22 (1.03-1.43)		0.20 (0.12-0.32)		0.51 (0.31-0.83)		0.56 (0.34-0.93)		0.57 (0.34-0.97)		0.59 (0.35-0.99)	
High CRF, >45 mL/min/kg	0.21 (0.17-0.25)		1.08 (0.88-1.34)		1.01 (0.81-1.24)		0.92 (0.74-1.15)		0.92 (0.72-1.16)		0.04 (0.02-0.12)		0.25 (0.09-0.70)		0.28 (0.10-0.81)		0.29 (0.10-0.86)		0.30 (0.10-0.87)	

Abbreviations: CRF, cardiorespiratory fitness; HPA, health profile assessment; HR, hazard ratio; NA, not applicable.

^a^ Model 1 adjusted for age and year of HPA. Model 2 for colon and prostate cancer, additionally adjusted for length of education, diet habits, comorbidity, and smoking. Model 2 for lung cancer, additionally adjusted for length of education, diet habits, comorbidity, and body mass index. Model 3 for colon and prostate cancer, additionally adjusted for body mass index. Model 3 for lung cancer, additionally adjusted for smoking.

## Results

A total of 177 709 men (mean [SD] age, 42 [11] years; range, 18-75 years) were included in the analyses. During a mean (SD) follow-up time of 9.6 (5.5) years, a total of 499 incident cases of colon cancer, 283 cases of lung cancer, and 1918 cases of prostate cancer occurred (Table 1). During the same mean follow-up time, there were 152 deaths due to colon cancer, 207 due to lung cancer, and 141 due to prostate cancer. Table 1 provides specific follow-up times for each outcome; eFigure 3, eFigure 4, and eFigure 5 in Supplement 1 provide cancer-specific cumulative incidence and survival according to the 4 groups of CRF stratification.

Adjusting for age, patients with incident cases of cancer had a lower mean (SE) estimated V̇o_2_max compared with those without cancer: 35.4 (0.4) vs 36.7 (0.1) mL/min/kg (*P* < .001) for colon cancer, 35.8 (0.5) vs 36.7 (0.1) mL/min/kg (*P* = .01) for lung cancer, and 37.6 (0.2) vs 36.7 (0.1) mL/min/kg (*P* < .001) for prostate cancer. Patients who died had lower mean (SE) CRF compared with those who did not die: 35.8 (0.7) vs 36.7 (0.1) mL/min/kg (*P* = .24) for colon cancer, 35.6 (0.6) vs 36.7 (0.1) mL/min/kg (*P* = .09) for lung cancer (although these results were not significant), and 34.8 (0.8) vs 36.7 (0.1) mL/min/kg (*P* = .01) for prostate cancer.

**Figure 1.**
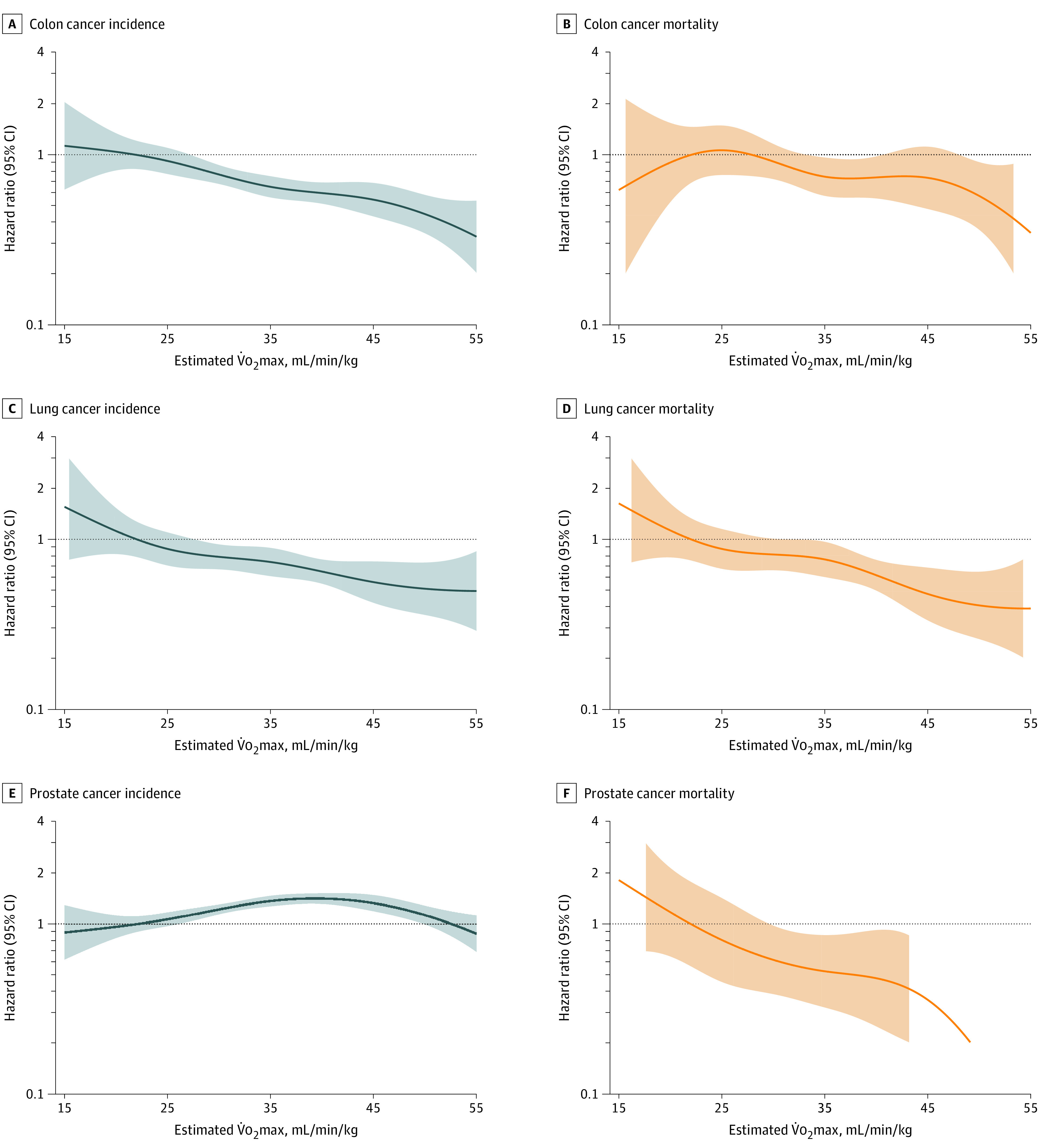
Association Between Cardiorespiratory Fitness and Incidence and Mortality in Colon Cancer, Lung Cancer, and Prostate Cancer Lines indicate hazard ratio; shaded area is 95% CI. Reference level set to 22 mL/min/kg and knots to 25, 35, and 45 mL/min/kg. V̇o_2_max indicates maximal oxygen consumption.

Higher estimated V̇o_2_max was associated with significantly lower risks of colon (HR, 0.98; 95% CI, 0.96-0.98) and lung (HR, 0.98; 95% CI, 0.96-0.99) cancer incidence, but with a higher risk of prostate cancer incidence (HR, 1.01; 1.00-1.01) (Figure 1). Higher estimated V̇o_2_max was associated with a lower risk of colon cancer death (HR, 0.98; 95% CI, 0.96-1.00) and a lower risk of death due to lung (HR, 0.97; 95% CI, 0.95-0.99) and prostate (HR, 0.95; 95% CI, 0.93-0.97) cancer.

After further adjusting for length of education, diet habits, comorbidity, and smoking for colon and prostate cancer, only colon cancer incidence and prostate cancer mortality remained significant for the moderate and high groups compared with those with very low CRF (Table 2, model 2). When adjusting for length of education, diet habits, comorbidity, and BMI for lung cancer, the associations remained significant for all groups compared with very low CRF (Table 2; model 2). After adjusting for BMI in model 3, moderate (HR, 0.72; 95% CI, 0.53-0.96) and high (HR, 0.63; 95% CI, 0.41-0.98) CRF were associated with a lower risk for colon cancer incidence compared with very low CRF. Furthermore, low (HR, 0.67; 95% CI, 0.45-1.00), moderate (HR, 0.57; 95% CI, 0.34-0.97), and high (HR, 0.29; 95% CI, 0.10-0.86) CRF were associated, in a dose-response manner, with lower prostate mortality, after additionally adjusting for BMI in model 3. After adjusting for smoking in model 3, the risk of death due to lung cancer was significantly lower only in the group with high CRF compared with the very low CRF group (HR, 0.41; 95% CI, 0.17-0.99).

**Figure 2.**
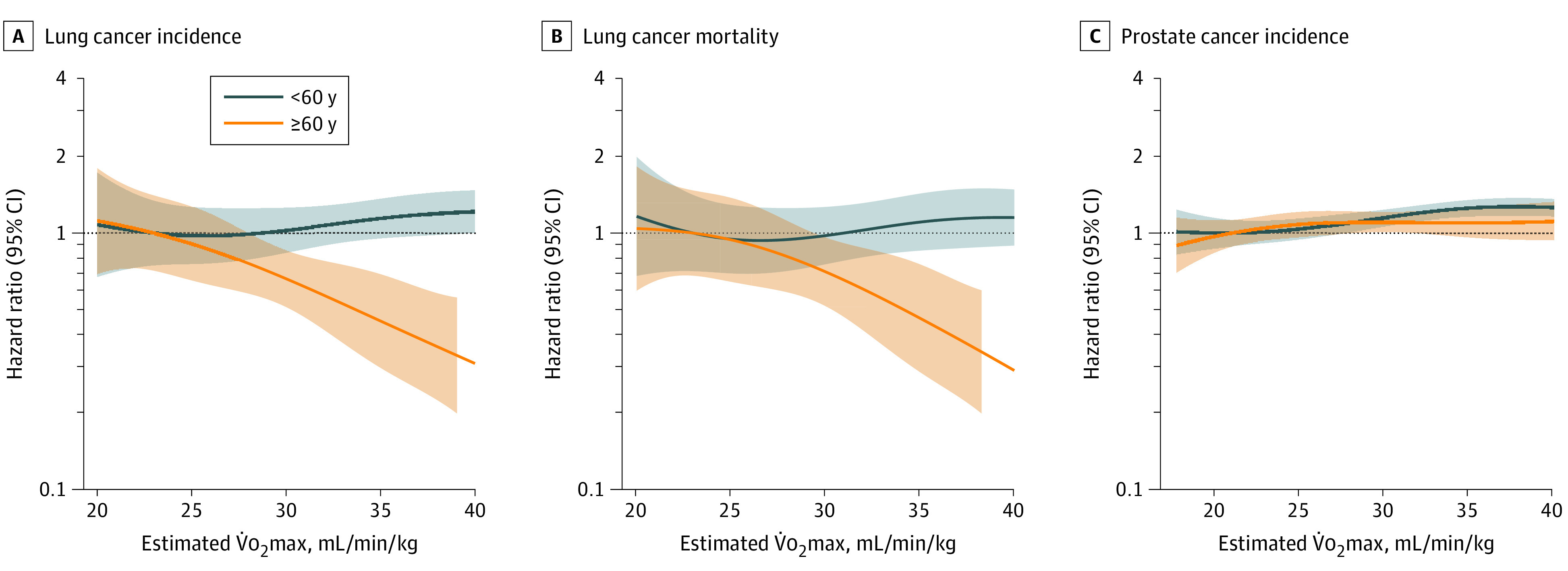
Age-Specific (<60 Years and ≥60 Years) Associations Between Cardiorespiratory Fitness and Lung Cancer Incidence and Mortality, and Prostate Cancer Incidence Lines indicate hazard ratio; shaded area is 95% CI. Reference level set to 22 mL/min/kg, and knots to 25 and 35 mL/min/kg. V̇o_2_max indicates maximal oxygen consumption.

Age modified the associations between CRF and risk of lung cancer incidence (HR, 0.99; 95% CI, 0.99-0.99; *P* = .02) and death (HR, 0.99; 95% CI, 0.99-0.99; *P* = .04), where lower risks with higher CRF were evident only in older participants (age, ≥60 years) (Figure 2). Age also modified the association between CRF and prostate cancer incidence (HR, 1.00; 95% CI, 1.00-1.00; *P* < .001), with a marginally higher risk for prostate cancer incidence for younger participants (age, <60 years) with increasing CRF.

Prevented fraction for the population, presented as theoretical prevented cases, of colon cancer incidence was 8% in the moderate CRF group and 4% in the high CRF group. For lung cancer mortality, the theoretically prevented death incidence was 4% in the high CRF group. For prostate cancer mortality, theoretically prevented death was 19% in the low CRF group, 14% in the moderate CRF group, and 7% in the high CRF group.

## Discussion

The main finding in this large study including 177 709 men was that higher CRF was associated with a lower risk for colon cancer incidence, lung cancer incidence and death, and prostate cancer death. Some of the findings were significant for colon cancer incidence and prostate cancer mortality after adjusting for lifestyle habits, comorbidities, and BMI, and after adjustment for lifestyle habits, comorbidities, and smoking for lung cancer mortality. After adjustment for age, an association between CRF and lung cancer incidence and mortality was evident only in older (age, ≥60 years) participants. The percentage of theoretically prevented cases of lung cancer and deaths from lung and prostate cancer, attributable to having higher than very low CRF, were between 4% and 19%.

The novelty of this study is in the analyses of the association between CRF and both incidence and mortality in the 3 most common cancers in men separately in a large sample of differing ages. In line with PA and prostate cancer studies, there is considerable inconsistency in findings on the association between CRF and the incidence of prostate cancer.^20,21^ While the results of the present study reflect the positive associations previously reported,^8,22,23^ others found a protective effect of CRF, albeit limited to participants younger than 60 years.^9^ A large Swedish study of 1.2 million adolescents followed up for 40 years reported an inverse association between CRF and cancer incidence of the most common cancers in men (prostate cancer included). However, the analyses were not conducted by cancer type, so the ability to detect any different or opposing associations may have been lost.^24^ The influence of potentially higher prostate cancer screening rates in those with higher CRF has been put forward as an explanation for the counterintuitive association between CRF and prostate cancer incidence found in this study and others.^8^ Reiter-Brennan et al^11^ reported that men with high CRF had a 28% higher risk of completing prostate cancer screening in comparison with men with low CRF. It is important to also consider that prostate cancer is the most heritable cancer, with a reported 58% of the variability in risk attributable to inherited risk factors,^25^ meaning that lifestyle factors may be less likely to influence prostate cancer incidence rates than other cancers prone to lifestyle-related cancer-causing agents.

Higher CRF conferred a lower risk of prostate and lung cancer death in the present study. Most existing studies have long reported that high CRF has protective outcomes on all-cancer mortality in men.^24,26,27^ While evidence for cancer-specific mortality is limited,^23,28^ both the results of the present study and a limited number of existing studies have reported the beneficial outcomes associated with higher CRF for lung-, colon-, and prostate-cancer–specific mortality.^10,28^

Associations remained after adjusting for BMI, for the moderate and high CRF groups for colon cancer incidence, and for the low, moderate, and high CRF groups for prostate cancer mortality. After further adjusting for smoking, associations were only significant for lung cancer mortality for the high CRF group. These associations have also been reported in other studies^20,29^ and highlight the benefits of CRF on cancer incidence and mortality for certain cancers. In the theoretical calculation of prevented cases, we noted that avoiding having very low CRF levels could have prevented 4% to 8% of all colon cancer cases, 4% of all deaths from lung cancer, and 4% to 19% of deaths from prostate cancer. Potential mechanisms to explain the benefits of higher CRF on cancer incidence and mortality include the association between CRF and systemic inflammation,^30^ abdominal obesity,^31^ dyslipidemia,^32^ and insulin sensitivity.^33^ Despite the increasing knowledge of these potential mechanisms, they are not yet fully understood, and more research is needed to confirm their role in the effect of CRF on cancer incidence and mortality.

The variation in age at baseline assessment in the present study presented the opportunity to examine whether associations between CRF and cancer risk differ over the life course. After adjustment for age, the lower risk with higher CRF for lung cancer incidence and death was evident only in participants 60 years or older at the time of the CRF assessment. While we controlled for smoking, one hypothesis could be that the detrimental effects of a greater number of years of long-term smoking on CRF in the older participants, even before a lung cancer diagnosis, may have led to a more likely association between the participants’ CRF and risk of lung cancer. The findings highlight the importance of considering a life course approach when examining CRF and cancer incidence and mortality.

The clinical implications of these findings further emphasize the importance of CRF for possibly reducing cancer incidence and mortality. We believe the implications are 2-fold. It is clinically relevant for the general public to understand the distinction that while physical activity and CRF are related, PA is the behavior (often measured through self-report) and CRF is the physiologic response (measured using objective assessments) within the body to the different doses and intensities of physical activity. It is important for the general public to understand that higher-intensity PA has greater effects on CRF and is likely to be more protective against the risk of developing and dying from certain cancers. In addition, the results of this study are important when communicating risk levels for people without cancer who have completed exercise tests to understand that CRF is not only important for cardiovascular disease risk, but also cancer risk.

### Strengths and Limitations

Strengths of this study include that it represents what we believe to be the largest cohort study of CRF and its association with both incidence and mortality in specific cancer types in men.

The present study has some limitations. One limitation was the voluntary participation and inclusion of only people who were employed. Moreover, submaximal tests only estimate V̇o_2_max; however, the submaximal protocol used has been reported to produce valid and reliable estimations of actual compared with directly measured V̇o_2_max.^34^ The significant genetic component of both CRF level and cancer risk must also be considered when interpreting the results. Ideally, analysis models for colorectal cancer incidence should include information on aspirin and statin use; however, information on medications was not available in the data sets. Despite the large initial cohort, the present study had a small number of cancer incident cases and deaths; this may have been due to the relatively short follow-up and should be considered when interpreting the results. Change in CRF could provide additional information, and the research group is currently working on these analyses. The question on smoking status was not optimal as it did not provide information on whether participants were current or past smokers or capture whether people’s smoking habits had changed over time. In addition, these findings may not generalizable for other cancer populations.

## Conclusions

In this prospective cohort study of 177 709 Swedish men, we found that moderate and high CRF were associated with a lower risk of colon cancer. Low, moderate, and high CRF were associated with lower risk of death due to prostate cancer, while only high CRF was associated with lower risk of death due to lung cancer. Age modified the results so that higher CRF was associated with a lower risk for lung cancer incidence and death only in older participants. If these findings can be supported with randomized clinical trials, CRF appears to have a potentially important role in reducing the risk of developing and dying from certain common cancers in men. Future research should consider examining exposures at different times in the life course and the associations between CRF and cancer incidence and mortality.

## Notes

Supplement 1. **eFigure 1.** Working Model of Possible Causal Pathways Leading to Cancer in Men **eFigure 2.** Flow Chart of Included and Excluded Participants **eFigure 3.** Cumulative Colon Cancer Incidence and Survival in Relation to Cardiorespiratory (CRF) Group **eFigure 4.** Cumulative Lung Cancer Incidence and Survival in Relation to Cardiorespiratory (CRF) Group **eFigure 5.** Cumulative Prostate Cancer Incidence and Survival in Relation to Cardiorespiratory (CRF) Group

Supplement 2.
Data Sharing Statement
