# Supplementary material for: Association Between Cardiorespiratory Fitness and Cancer Incidence and Cancer-Specific Mortality of Colon, Lung, and Prostate Cancer Among Swedish Men
Source: JAMA Netw Open. 2023 Jun 29;6(6):e2321102. doi: 10.1001/jamanetworkopen.2023.21102 (PMC10311389; doi:10.1001/jamanetworkopen.2023.21102)
Supplement: Supplement 1. — eFigure 1. Working Model of Possible Causal Pathways Leading to Cancer in Men eFigure 2. Flow Chart of Included and Excluded Participants eFigure 3. Cumulative Colon Cancer Incidence and Survival in Relation to Cardiorespiratory (CRF) Group eFigure 4. Cumulative Lung Cancer Incidence and Survival in Relation to Cardiorespiratory (CRF) Group eFigure 5. Cumulative Prostate Cancer Incidence and Survival in Relation to Cardiorespiratory (CRF) Group [file jamanetwopen-e2321102-s001.pdf]

Supplementary Online Content

Ekblom-Bak E, Bojsen-Møller E, Wallin P, et al. Association between cardiorespiratory fitness and cancer incidence and cancer-specific mortality of colon, lung, and prostate cancer among Swedish men. *JAMA Netw Open*. 2023;6(6):e2321102. doi:10.1001/jamanetworkopen.2023.21102

eFigure 1. Working Model of Possible Causal Pathways Leading to Cancer in Men

eFigure 2. Flow Chart of Included and Excluded Participants

eFigure 3. Cumulative Colon Cancer Incidence and Survival in Relation to Cardiorespiratory (CRF) Group

eFigure 4. Cumulative Lung Cancer Incidence and Survival in Relation to Cardiorespiratory (CRF) Group

eFigure 5. Cumulative Prostate Cancer Incidence and Survival in Relation to Cardiorespiratory (CRF) Group

This supplementary material has been provided by the authors to give readers additional information about their work.

© 2023 Ekblom-Bak E et al. *JAMA Network Open.*

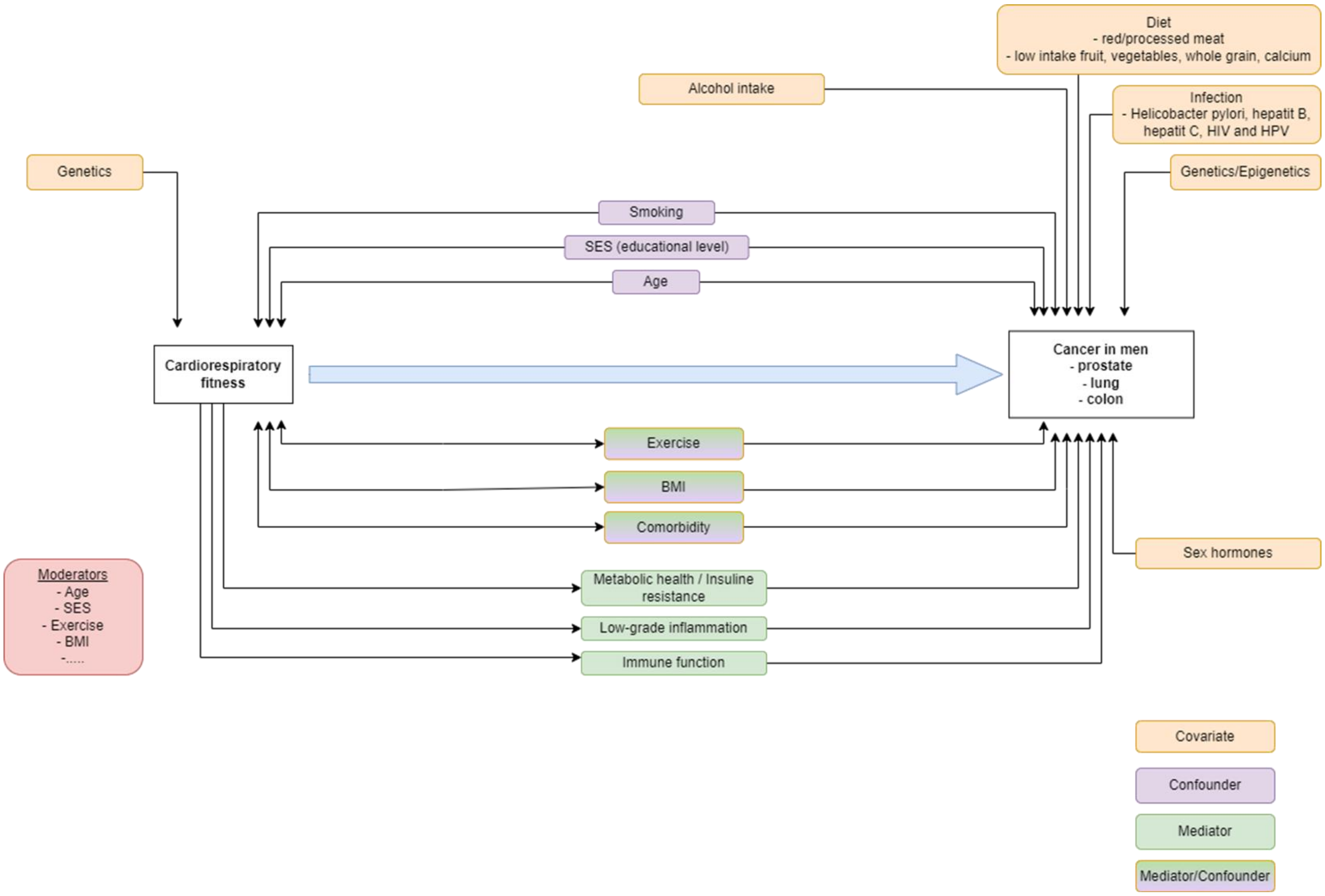

eFigure 1. **Working model of possible causal pathways leading to cancer in men.**

© 2023 Ekblom-Bak E et al. *JAMA Network Open.*

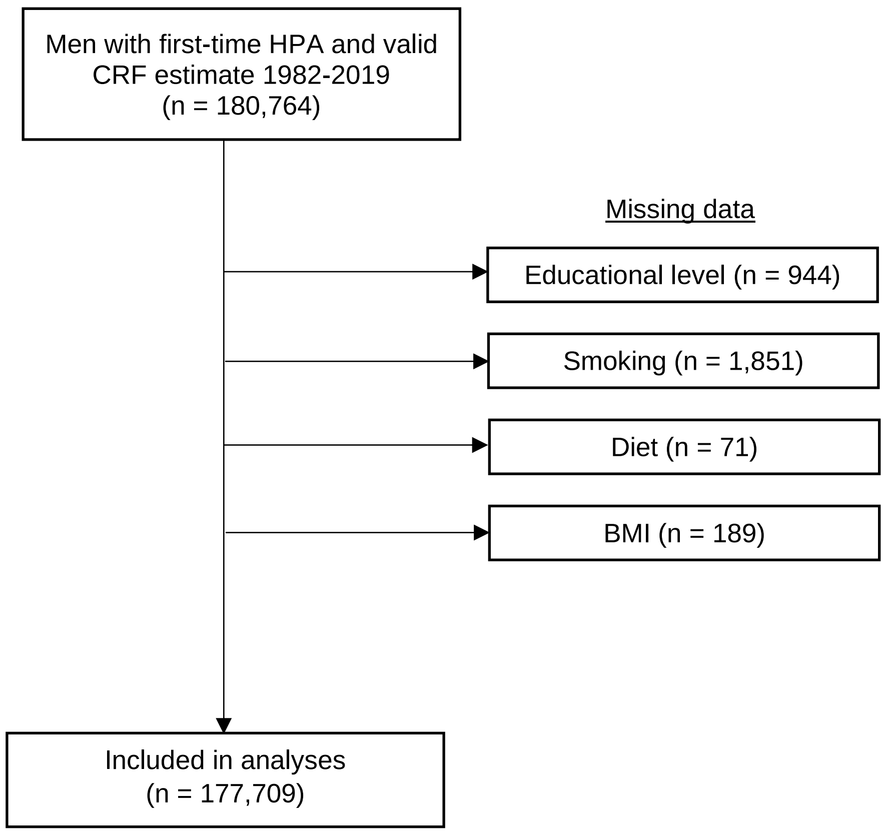

eFigure 2. **Flow chart of included and excluded participants**

© 2023 Ekblom-Bak E et al. *JAMA Network Open.*

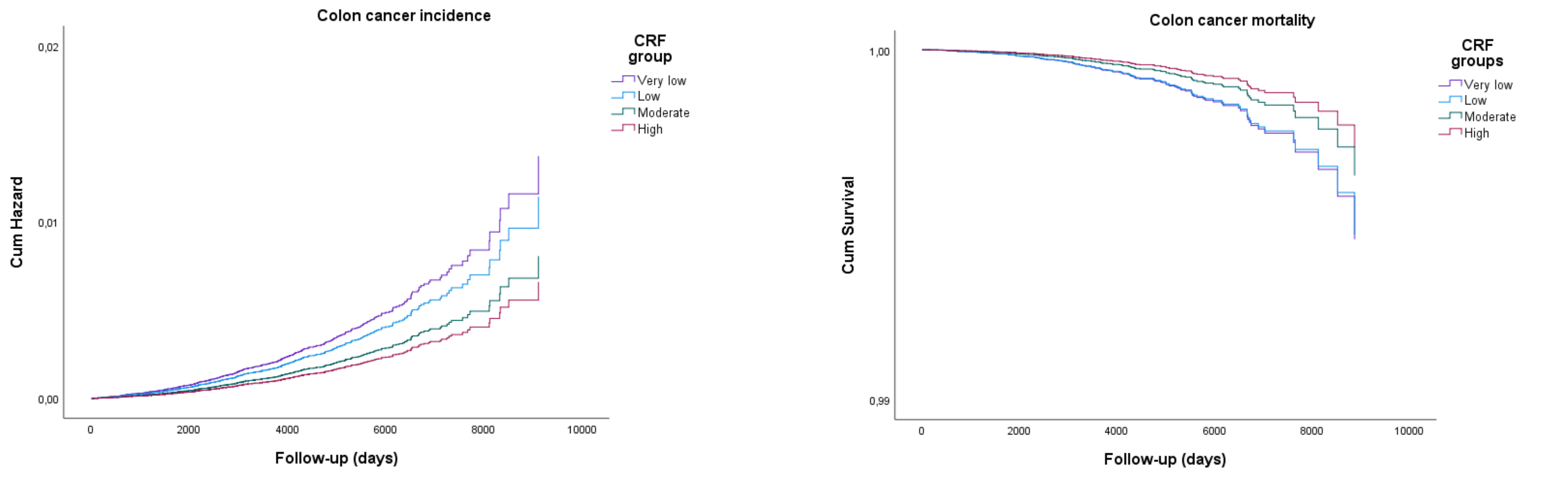

eFigure 3. **Cumulative colon cancer incidence and survival in relation to cardiorespiratory (CRF) group.** Very low CRF (≤25 ml/min(kg), Low CRF (>25-35 ml/min/kg), Moderate CRF (>35-45 ml/min/kg), High CRF (>45 ml/min/kg). Adjusted for year of HPA and age at HPA.

© 2023 Ekblom-Bak E et al. *JAMA Network Open.*

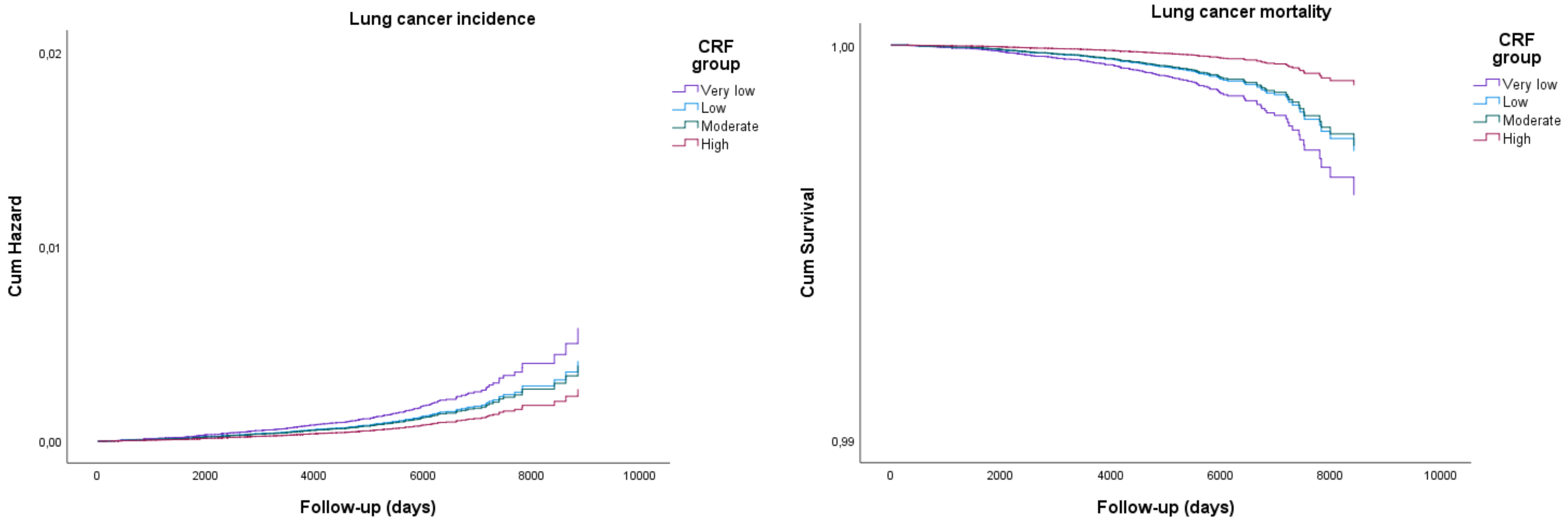

eFigure 4. **Cumulative lung cancer incidence and survival in relation to cardiorespiratory (CRF) group.** Very low CRF (≤25 ml/min(kg), Low CRF (>25-35 ml/min/kg), Moderate CRF (>35-45 ml/min/kg), High CRF (>45 ml/min/kg). Adjusted for year of HPA and age at HPA.

© 2023 Ekblom-Bak E et al. *JAMA Network Open.*

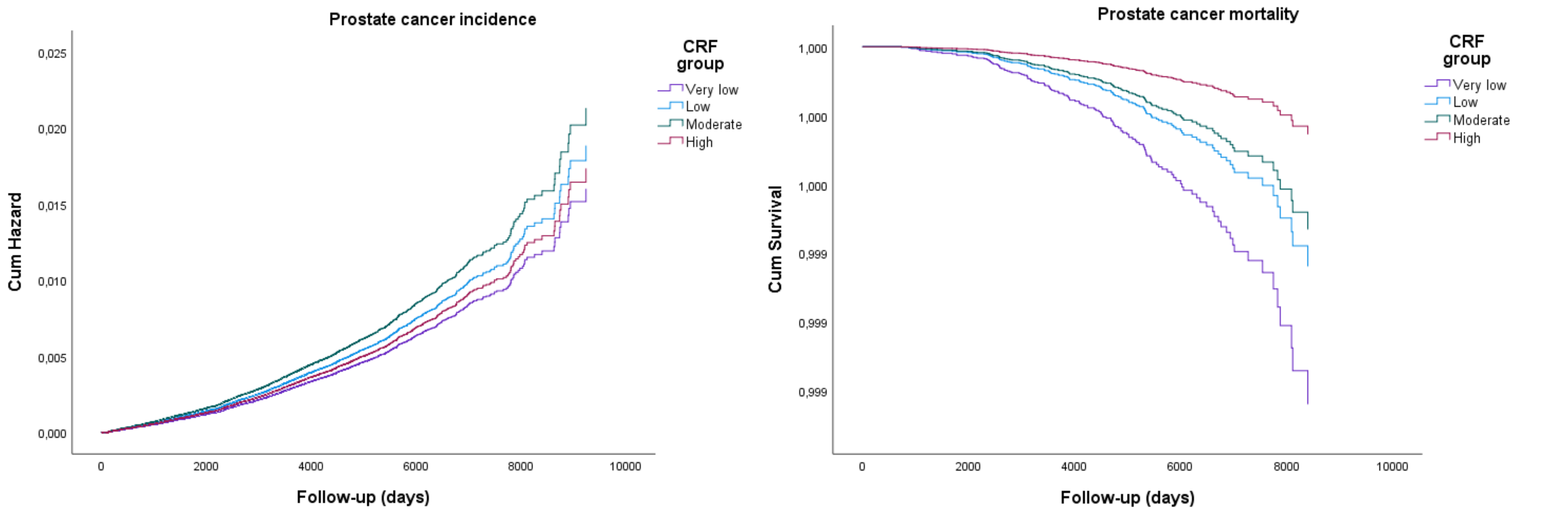

eFigure 5. **Cumulative prostate cancer incidence and survival in relation to cardiorespiratory (CRF) group.** Very low CRF (≤25 ml/min(kg), Low CRF (>25-35 ml/min/kg), Moderate CRF (>35-45 ml/min/kg), High CRF (>45 ml/min/kg). Adjusted for year of HPA and age at HPA.

© 2023 Ekblom-Bak E et al. *JAMA Network Open.*
