# Supplementary material for: Association Between Cardiorespiratory Fitness and Cancer Incidence and Cancer-Specific Mortality of Colon, Lung, and Prostate Cancer Among Swedish Men
Source: JAMA Netw Open. 2023 Jun 29;6(6):e2321102. doi: 10.1001/jamanetworkopen.2023.21102 (PMC10311389; doi:10.1001/jamanetworkopen.2023.21102)
Supplement: Supplement 2. — Data Sharing Statement [file jamanetwopen-e2321102-s002.pdf]

Data Sharing Statement

Ekblom-Bak. Association Between Cardiorespiratory Fitness and Cancer Incidence and Cancer-Specific Mortality of Colon, Lung, and Prostate Cancer Among Swedish Men. *JAMA Netw Open*. Published June 29, 2023. doi:10.1001/jamanetworkopen.2023.21102

Data
Data available: No

Additional Information
Explanation for why data not available: The data underlying the findings in our study are not publicly available because the original approval from the Regional ethics board and the informed consent form did not include such direct, free access to the data. Data are owned by, and can be requested from, the HPI Health Profile Institute at support@hpihealth.se.
